# Prediction models for early neurological deterioration in patients with acute ischemic stroke: a systematic review and critical appraisal

**DOI:** 10.3389/fneur.2026.1737871

**Published:** 2026-02-24

**Authors:** Xiangyi Zheng, Miaomiao Zhao, Zhaowen Yang, Ligaoge Kang, Ruxue Li, Ying Gao, Genming Zhang, Xinxing Lai

**Affiliations:** 1 Department of Neurology, Dongzhimen Hospital, Beijing University of Chinese Medicine, Beijing, China; 2 Fangshan Hospital, Beijing University of Chinese Medicine, Beijing, China; 3 School of Nursing, Beijing University of Chinese Medicine, Beijing, China; 4 Institute for Brain Disorders, Beijing University of Chinese Medicine, Beijing, China

**Keywords:** acute ischemic stroke, critical appraisal, early neurological deterioration, prediction model, systematic review

## Abstract

**Background:**

Despite the proliferation of risk prediction models for early neurological deterioration (END) in patients with acute ischemic stroke (AIS), significant uncertainties persist regarding their methodological rigor and clinical applicability.

**Objective:**

To systematically review and critically evaluate published prediction models for END in patients with AIS.

**Methods:**

PubMed, Embase, Scopus, and the Cochrane Library were searched from inception to March 26, 2025. Data extraction was conducted using a standardized data extraction form by two independent reviewers based on the recommendations in the CHecklist for critical Appraisal and data extraction for systematic Reviews of prediction Modelling Studies (CHARMS). The Prediction model Risk Of Bias ASsessment Tool (PROBAST) checklist was used to assess the risk of bias and applicability. A qualitative synthesis was carried out to summarize the main characteristics of the included studies and constructed models.

**Results:**

A total of 3,682 studies were retrieved, and 45 prediction models from 23 studies were included. Logistic regression and machine learning were utilized to establish END risk prediction models. The reported incidence of END in AIS patients varied from 6.6 to 43.7%, depending on the definition and study population. The most frequently used predictors were baseline National Institutes of Health Stroke Scale score and systolic blood pressure. The model’s discrimination performance, quantified by the area under the curve or concordance statistic, showed remarkable heterogeneity in predictive accuracy across studies. Critically, all included studies were assessed as having a high risk of bias, mainly owing to inappropriate data sources and poor reporting of the analysis domain. Concerns regarding applicability were generally low across studies.

**Conclusion:**

This systematic review provides a comprehensive mapping and critical assessment of existing END prediction models in AIS. The findings reveal a critical gap that current models exhibit high risk of bias, limiting their reliability for clinical adoption. Future research should prioritize prospective model development and validation with pre-specified protocols, rigorous adherence to methodological standards such as the TRIPOD guidelines, adequate sample size estimations, robust external validation, as well as the update and clinical utility of existing predictive models.

**Systematic review registration:**

PROSPERO, identifier (CRD42025643096).

## 1 Introduction

Stroke ranks globally as the third leading cause of death and the fourth contributor to disability-adjusted life-years (DALYs), constituting a major public health concern that increasingly strains healthcare systems (1). Ischemic stroke accounts for approximately 70% of all incident strokes (2). Timely intervention is pivotal for optimizing clinical outcomes in acute ischemic stroke (AIS) patients. The primary therapeutic objectives focus on rapid reperfusion and revascularization of the ischemic penumbra, thereby salvaging viable brain tissue and minimizing neurological deficits (3). Although functional outcomes for AIS patients are theoretically expected to improve following standard treatment, a subset of patients may fail to regain their functional level at admission (i.e., pre-treatment status) or even develop aggravated neurological deficits within hours to days after symptom onset—this phenomenon is termed early neurological deterioration (END) (4, 5). The reported incidence of END ranges from 5 to 40% across studies, primarily due to substantial variations in diagnostic criteria (6). END demonstrates a robust correlation with unfavorable clinical outcomes, manifesting as poor functional recovery at 3 months and elevated mortality rates (6–9). To ease healthcare strain while enhancing patient outcomes, reliable prediction of END in AIS remains important to inform decisions regarding pre-emptive interventions, rapid diagnosis, risk stratification, and long-term treatment.

Prediction models are multivariable tools that estimate an individual’s probability of a current or future health outcome based on baseline predictors (10–12). In recent years, numerous studies have attempted to develop or validate prediction models for estimating the risk of END in AIS patients. Despite this surge in interest, the methodological rigor of these models and their applicability across diverse clinical settings remain unclear. Furthermore, competing prediction models often exist for the same outcome or target population, leaving clinicians uncertain about which model to adopt, particularly regarding which subgroups or healthcare contexts these tools best suit. To address these uncertainties, a systematic review will be conducted to critically appraise the methodological quality, predictive performance, and clinical applicability of available prediction models for END in AIS. This synthesis will clarify strengths and limitations of existing models, and provide robust guidance for future research on early risk stratification and patient outcomes in AIS.

## 2 Materials and methods

### 2.1 Study design

This systematic review was conducted in accordance with the Preferred Reporting Items for Systematic reviews and Meta-Analysis (PRISMA) (13) and the Transparent Reporting of a multivariable prediction model for Individual Prognosis Or Diagnosis (TRIPOD) (12) checklists. The study protocol was registered on the International Prospective Register of Systematic Reviews PROSPERO (CRD42025643096).

We also utilized the PICOTS system which was recommended by the CHecklist for critical Appraisal and data extraction for systematic Reviews of prediction Modelling Studies (CHARMS) checklist (14) to guide the framing of the review aim, search strategy, and study inclusion and exclusion criteria. The key items are described in Table 1.

**Table 1:** Key items for framing the aim, search strategy, and study inclusion and exclusion criteria for systematic review.

Item	Definition
Population	AIS patients
Intervention model	Development or validation or updating of the END prediction models for AIS patients
Comparator	Not applicable
Outcome	The outcome focused on END rather than its subgroups
Timing	The outcome was predicted after evaluating demographic characteristics, medical history, clinical information, and laboratory test results at admission
Setting	No limitation, inpatients or outpatients

AIS, acute ischemic stroke; END, early neurological deterioration.

### 2.2 Literature search strategy

PubMed, Embase, Scopus, and the Cochrane Library were systematically searched for relevant articles published in English from inception to March 26, 2025. The following keywords were used to conduct a basic search: “ischemic stroke,” “acute ischemic stroke”, “ischemic encephalopathy”, “cerebral infarction”, “nomogram”, “prediction model”, “risk score”, “neurological deterioration”, “neurological worsening” and “neurological decline.” The eligibility of the retrieved studies and any probable missing pertinent research were further determined by manually reviewing the reference lists of the studies. Specific details regarding the strategies are listed in the Supplementary material.

### 2.3 Inclusion and exclusion criteria

The inclusion criteria were as follows: (1) Study type: We considered observational studies; (2) Study participants: We involved participants aged 18 years or older diagnosed with AIS, referring to any authoritative or recognized diagnostic criteria. All cases were confirmed by brain Computed Tomography (CT) or Magnetic Resonance Imaging (MRI); (3) Study content: The purpose of the study was to develop, validate or update the END prediction models for AIS patients; (4) Primary outcome to be predicted: The occurrence of END.

The exclusion criteria were as follows: (1) Articles only studied independent risk factors without prediction model development or validation; (2) Literature not written in English; (3) Conference abstracts, review articles, letters, comments, editorials, preprints and errata; (4) Studies without full text available; (5) Duplicated publications: For the same research study published in multiple versions (including different journals, online preprints vs. formal published versions, full-text vs. abstract duplicates, etc.), only one most comprehensive and complete version is retained, and other duplicate versions are excluded.

### 2.4 Literature selection and data extraction

Two investigators (XZ, MZ) independently reviewed the titles and abstracts to select literature for further screening. After removal of irrelevant literature, the full text was scrutinized for inclusion in the systematic review. Disagreements were resolved through discussion or consultation by a third investigator (XL).

Data extraction was performed using a standardized data extraction form by two independent reviewers (XZ, MZ) in accordance with the CHARMS checklist. The data extracted from the included studies concerned: the first author, year of publication, country, study design, the target population, data source, outcome to be predicted, criteria of outcome, sample size, sample of models, the modeling method used, the handling of missing data, the handling of continuous data, the selection of variables, the validation method of models (internal and/or external validation), predictors included in the final model, the model performance (discrimination, calibration, and clinical utility), and the model presentation.

### 2.5 Quality assessment

The risk of bias (ROB) and applicability of the included studies were assessed by the Prediction model Risk Of Bias ASsessment Tool (PROBAST) (11). The ROB assessment contains 20 signaling questions categorized into four domains, namely participants, predictors, outcome and analysis. Each signaling question can be answered as yes, probably yes, no, probably no, or no information. Yes or probably yes indicates low ROB, while no or probably no indicates high ROB. If at least one signaling question in a domain is answered as no or probably no, that domain should be considered at high ROB. Only when all domains are judged as low ROB, the overall bias can be considered at low ROB. Applicability assessment involved three domains, namely participants, predictors, and outcome. The applicability assessment employs a methodological framework identical to that of the ROB evaluation.

### 2.6 Data synthesis

We chose not to conduct a meta-analysis given the marked heterogeneity observed in the selected population and the model characteristics. Instead, a descriptive analysis was carried out to summarize the basic characteristics of the included studies and constructed models.

## 3 Results

### 3.1 Literature search results

**Figure 1:**
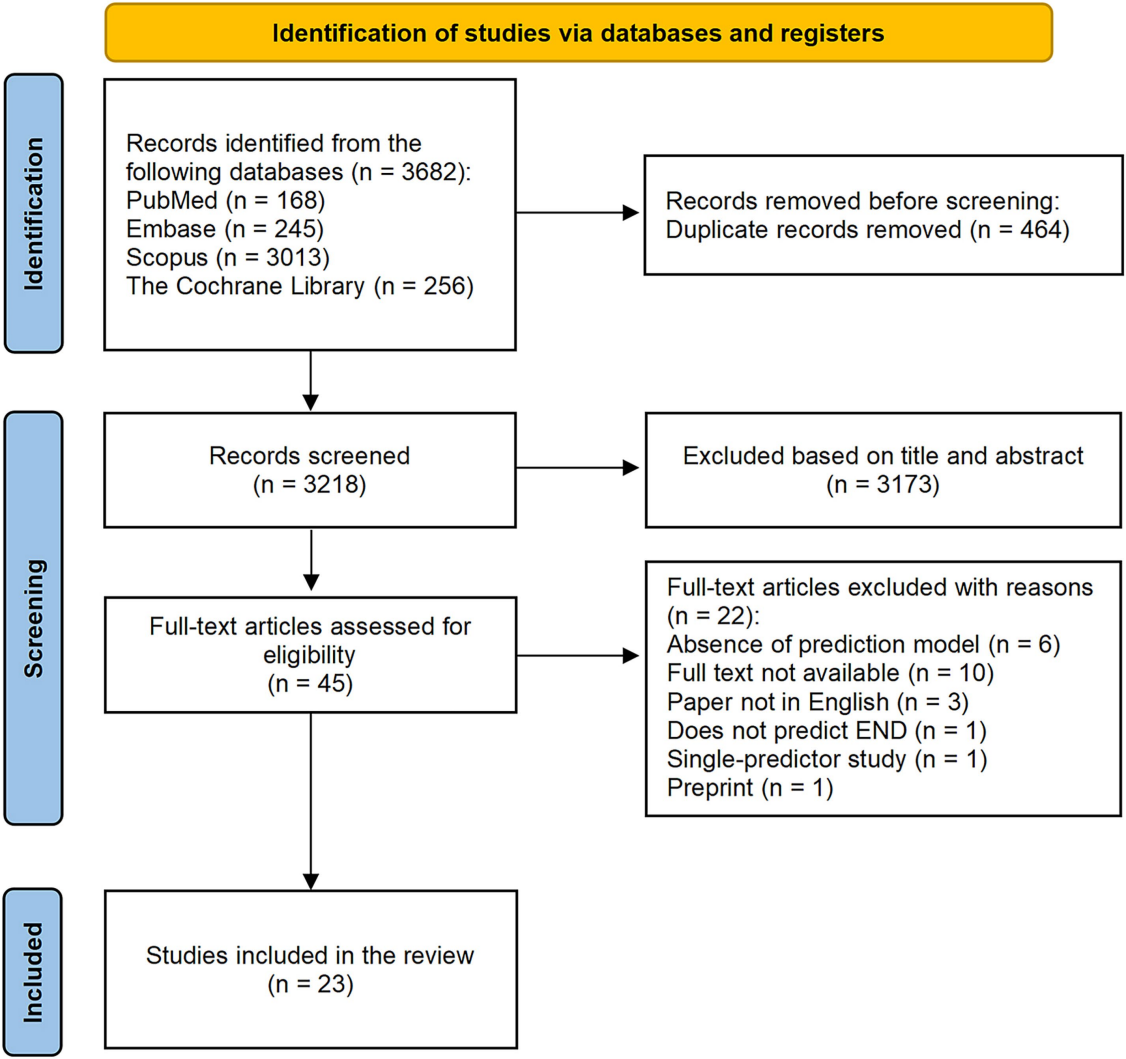
Flowchart of literature screening process.

Our preliminary search yielded 3,683 articles. After the removal of duplicate studies, 3,218 articles remained for screening. After screening both titles and abstracts, 3,173 studies were eliminated, and 45 full-text articles were further assessed for eligibility. We excluded 10 studies that lacked accessible data, six studies that did not establish prediction models, three studies that were not published in English, one study that did not undergo peer preview, one study that had less than two predictors, and one study that had outcomes limited to subgroups. Ultimately, 23 studies with 45 models were included in our systematic review. The literature screening process is provided in Figure 1.

### 3.2 Study characteristics

We included a total of 23 studies (15–37) in this review. Of the included studies, two combined a retrospective and prospective design for data collection (32, 34), three were prospective (18, 30, 36), and 18 were retrospective. They were released from 2017 to 2024. There were 19 studies carried out in China, and one each in Japan (37), Korea (33), and France (36). One study was conducted in both France and Switzerland (32). Twelve studies had a multicenter design (16, 17, 20, 22, 24–26, 32, 34, 36, 37), while the other were conducted in single centers. Regarding the research subjects, four studies included particular categories of AIS patients (21, 22, 32, 33), whereas nine studies focused on AIS patients receiving intravenous thrombolysis (IVT) (15, 17, 18, 20, 23, 24, 26, 27, 29), two on AIS patients receiving mechanical thrombectomy (MT) (25, 28), and one on AIS patients with endovascular treatment (EVT) (36). Each model study has its own set of outcome diagnostic criteria. Eight studies were evaluated by the increase of NIHSS total score ≥2 within 7 days of admission (16, 21, 22, 30, 31, 34, 35, 37), eight articles involving IVT patients concentrated on the changes of the NIHSS score within 24 to 72 h after thrombolysis (15, 17, 18, 20, 23, 26, 27, 32). The sample sizes ranged from 163 to 9,141 participants across the studies. The basic characteristics of the included studies are summarized in Table 2.

**Table 2:** Basic characteristics of included studies.

First author, publication year	Country	Study design	Populations	Criteria for END	Data source	Main endpoint	END cases/sample size	Number of models
Jiang Zhuangzhuang, 2024 (21)	China	Retrospective	BAD-related AIS patients with LSA territory infarction	The NIHSS total score increases by ≥2 within 7 days of admission	Single center	END	80/380 (21.6%)	9
Wang Jia, 2024 (22)	China	Retrospective	API patients	The NIHSS total score increases by ≥2 or the motor ability of NIHSS increases by ≥1 within 7 days of admission	Multi-center	END	108/544 (19.9%)	3
Luo Bang, 2024 (15)	China	Retrospective	AIS patients with IVT therapy	The NIHSS total score increases by ≥4 or the NIHSS individual score increases by ≥2 within 24 h after IVT treatment	Single center	END	33/217 (15.2%)	1
Zhou Yang, 2024 (16)	China	Retrospective	AIS patients	The NIHSS total score increases by ≥2 within 7 days of admission	Multi-center	END	140/1993 (7.0%)	1
Wen Rui, 2024 (17)	China	Retrospective	AIS patients with IVT therapy	The NIHSS score increases by ≥4 within 24 h after IVT treatment	Multi-center	END	1766/9141 (19.3%)	5
Li Ning, 2024 (20)	China	Retrospective	AIS patients with IVT therapy	The NIHSS score increases by ≥2 within 24–36 h after IVT treatment	Multi-center	END	−/531	1
Zhu Bifeng, 2024 (18)	China	Prospective	AIS patients with IVT therapy	The NIHSS total score increases by ≥2, the motor power score increases by ≥1, or the clinical symptoms fluctuation, within 48 h after IVT treatment	Single center	END	66/211 (31.3%)	1
Qiu Kai, 2024 (19)	China	Retrospective	AIS patients	The NIHSS score increases by ≥4 within 24 h	Multi-center	END	44/248 (17.7%)	1
Tian Tian, 2023 (23)	China	Retrospective	AIS patients with IVT therapy	The NIHSS score increases by ≥4 within 24 h after IVT treatment	Single center	END	88/426 (20.7%)	1
Jin Huijuan, 2023 (24)	China	Retrospective	AIS patients with IVT therapy	The NIHSS score increases >4 or death within 24 h of stroke onset.	Multi-center	END	101/1213 (8.3%)	1
Wu Kongyuan, 2023 (25)	China	Retrospective	AIS patients with MT therapy	The NIHSS score increases ≥2 within 72 h of admission	Multi-center	END	269/1007 (26.7%)	1
Yang Huan, 2023 (26)	China	Retrospective	AIS patients with IVT therapy	The NIHSS score increases ≥2 within 72 h of admission	Single center	END	99/704 (14.1%)	2
Jin Mengzhi, 2023 (27)	China	Retrospective	AIS patients with IVT therapy	The NIHSS score increases ≥2 within 24 h after IVT treatment	Single center	END	41/195 (21.0%)	2
Yang Tongtong, 2023 (28)	China	Retrospective	AIS patients with MT therapy	The NIHSS score increases by ≥4 from baseline to 24 h of the stroke event	Multi-center	END	213/1218 (17.5%)	4
Wang Mei, 2023 (29)	China	Retrospective	AIS patients with IVT therapy	The NIHSS score increases by ≥4, or death within 24 h after IVT treatment	Single center	END	90/321 (28.0%)	1
Wang Jia, 2022 (30)	China	Prospective	AIS patients	The NIHSS total score increases by ≥2 within 7 days of admission	Single center	END	164/375 (43.7%)	1
Xie Xiaohua, 2021 (31)	China	Retrospective	AIS patients	The NIHSS total score increases by ≥2 within 7 days of admission	Single center	END	64/391 (16.4%)	1
Seners Pierre, 2021 (32)	France and Switzerland	Retrospective and Prospective	Minor stroke and LVO patients with IVT therapy	The NIHSS score increases by ≥4 within 24 h after IVT treatment	Multi-center	END	126/1076 (11.7%)	1
Sung Sang Min, 2020 (33)	Korea	Retrospective	Acute minor ischemic stroke patients	Any worsening of neurological deficits within 3 days after admission.	Single center	END	78/739 (10.6%)	4
Gong Pengyu, 2020 (34)	China	Retrospective and Prospective	AIS patients	The NIHSS total score increases by ≥2 within 7 days of admission	Multi-center	END	430/2707 (15.9%)	1
Xu Yicheng, 2020 (35)	China	Retrospective	AIS patients	The NIHSS total score increases by ≥2, the motor power score increases by ≥1, or the clinical symptoms fluctuation, compared with baseline from baseline during the first 7 days after admission	Single center	END	90/354 (25.4%)	1
Girot Jean-Baptiste, 2020 (36)	France	Prospective	AIS patients with EVT therapy	The NIHSS score increases by ≥4 or death within 24 h of onset	Multi-center	END	128/1925 (6.6%)	1
Miyamoto Nobukazu, 2017 (37)	Japan	Retrospective	AIS patients	The NIHSS total score increases by ≥4 within 7 days of admission	Multi-center	END	112/934 (12.0%)	1

“-”, not reported; AIS, acute ischemic stroke; API, acute pontine infarction; BAD, branch atheromatous disease; END, early neurological deterioration; EVT, endovascular treatment; IVT, intravenous thrombolysis; LSA, lenticulostriate artery; LVO, large vessel occlusion; MT, mechanical thrombectomy; NIHSS, National Institutes of Health Stroke Scale.

### 3.3 Model development and validation

Among the included studies, the majority not only developed but also validated their models. Nevertheless, three studies merely developed models without performing validation (31, 33, 36). Meanwhile, one study verified the performance of the pre-existing models (35). Most of the studies utilized logistic regression to develop prediction models (n = 18, 78.3%). In contrast, four implemented various machine learning methods to estimate the risk of END (17, 21, 28, 33). The algorithms employed across the studies can be categorized as follows: Support Vector Machine (SVM), Logistic Regression (LR), Artificial Neural Network (ANN), Gradient Boosting Machine (GBM), Partial Least Squares (PLS), Naive Bayes Classifier (NBC), Bagging Trees and Random Forest (RF). The most frequently used predictors across the models were the National Institutes of Health Stroke Scale (NIHSS) score at admission and Systolic Blood Pressure (SBP), which appeared in 15 and 13 models, respectively. Other commonly used predictors included age, Neutrophil to Lymphocyte Ratio (NLR), Atrial Fibrillation (AF), and blood glucose. Moreover, some studies incorporated neuroimaging features derived from radiomic analysis into their prediction models (22).

**Figure 2:**
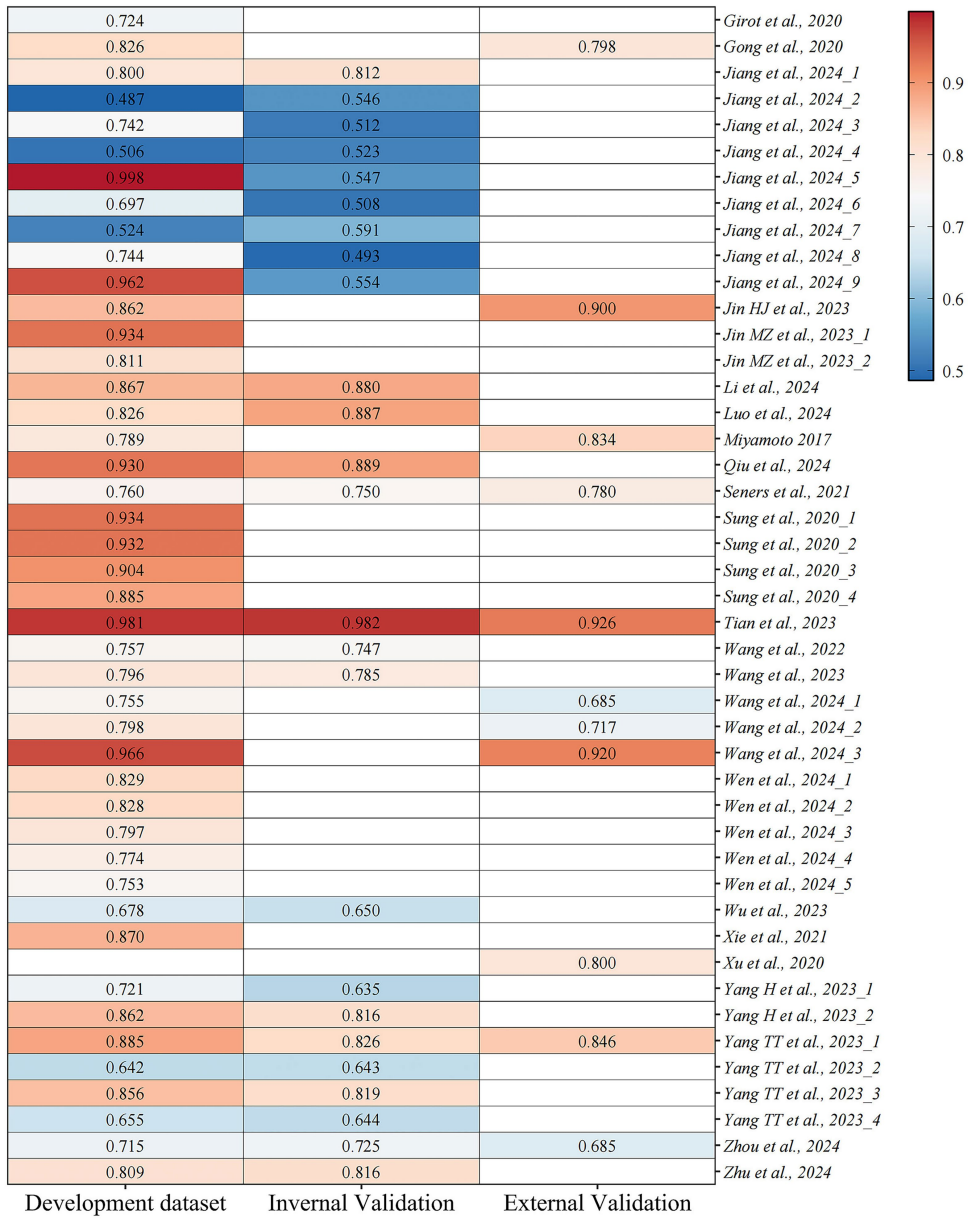
The areas under the curve (AUCs) or concordance statistics (C-statistics) among models.

Of 45 models included, most models were validated internally or externally. Specially, 20 models performed internal validation (15, 18–21, 25–27, 29, 30), six conducted external validation only (22, 24, 34, 37), and 12 contained both internal and external validation (16, 17, 23, 28, 32). The remaining six models were not validated after development (31, 33, 36). In addition, some researchers did not develop new prediction models but instead investigated the utility of the WORSEN score in China (35). Most studies quantified discrimination with the areas under the curve (AUC) or concordance statistics (C-statistics). The reported AUCs or C-statistics ranged from 0.487 to 0.998 in the derivation set and from 0.493 to 0.982 in the validation set. Detailed AUCs or C-statistics are shown in Figure 2. Currently, risk prediction models primarily consist of nomograms, equations, and tables. The majority of the prediction models incorporated in our study are nomograms. Calibration was reported in most models and generally demonstrated good performance. Researchers assessed calibration using the Hosmer-Lemeshow test, Brier scores, and calibration plots, with calibration plots being the most frequently employed method. Furthermore, 13 studies performed decision curve analysis (DCA). Further details are displayed in Table 3.

**Table 3:** Overview of the information of the included prediction models.

First author, publication year	Purpose	Modelling method	Missing data handling	Continuous variable processing method	Variables selection	Validation method	Final predictors	Model performance	Model calibration	Model presentation	Best performance model	Clinical utility
Jiang Zhuangzhuang, 2024 (21)	Model development and validation	LR, SVM, GBM, ANN, TG, PLS, NNET, Bayes, RF	Complete-case analysis	Continuous variable	LASSO regression and backward stepwise selection	Random split	Systolic pressure, stroke history, conglomerated beads sign, parent artery stenosis, MCA shape	A: LR: 0.800 (0.739–0.861) SVM: 0.487 (0.403–0.572) GBM: 0.742 (0.673–0.810) ANN: 0.506 (0.489–0.522) TG: 0.998 (0.996–1.000) PLS: 0.697 (0.624–0.771) NNET: 0.524 (0.491–0.558) Bayes: 0.744 (0.677–0.810) RF: 0.962 (0.943–0.982) B: LR: 0.812 (0.712–0.912) SVM: 0.546 (0.394–0.698) GBM: 0.512 (0.345–0.679) ANN: 0.523 (0.467–0.579) TG: 0.547 (0.407–0.688) PLS: 0.508 (0.358–0.658) NNET: 0.591 (0.470–0.712) Bayes: 0.493 (0.333–0.653) RF: 0.554 (0.423–0.685)	H-L and calibration plot	Nomogram	LR	DCA
Wang Jia, 2024 (22)	Model development and validation	LR	Complete-case analysis	Continuous variable	Univariate *p*-value	External validation	Radiomics signature: rad-score Clinical model: age, initial SBP, initial NIHSS, TG Clinical-radiomics model: age, initial SBP, initial NIHSS, TG, rad-score	Radiomics signature: A: 0.755 (0.693–0.817) C: 0.685 (0.594–0.776) Clinical model: A: 0.798 C: 0.717 Clinical-radiomics model: A: 0.966 (0.947–0.985) C: 0.920 (0.873–0.967)	H-L and calibration plot	Nomogram	—	DCA
Luo Bang, 2024 (15)	Model development and validation	LR	Complete-case analysis	Continuous variable	Univariate *p*-value	Random split	Smoking, NIHSS, NLR, HCY	A: 0.826 (0.719–0.932) B: 0.887 (0.763–1.000)	Calibration plot	Nomogram	—	DCA
Zhou Yang, 2024 (16)	Model development and validation	LR	Complete-case analysis	Continuous variable	LASSO regression	Random split and external validation	CAD, SBP, neutrophils, lymphocytes, TBil, UA, LDL	A: 0.715 (0.648–0.782) B: 0.725 (0.631–0.820) C1: 0.685 (0.541–0.829) C2: 0.673 (0.545–0.800)	H-L and calibration plot	Nomogram	—	DCA
Wen Rui, 2024 (17)	Model development and validation	LASSO logistic regression, SVM, RF, GBDT, MLP	Multiple imputations	Continuous variable	—	Random split and external validation	Gender, age, post awakening stroke, in hospital stroke, BMI, SBP, DBP, admission mRS score, admission NIHSS score, swallowing function score, ONT, TOAST classification, thrombolytic drug, antiplatelet therapy, anticoagulation therapy	LASSO regression: 0.829 (0.799–0.86) MLP: 0.828 (0.799–0.858) RF: 0.797 (0.764–0.829) GBDT: 0.774 (0.741–0.808) SVM: 0.753 (0.711–0.795)	—	—	LASSO regression	DCA
Li Ning, 2024 (20)	Model development and validation	LR	Complete-case analysis	Categorical variables	LASSO regression	Random split	Stroke history, BMI, age, OTT, glucose, SII	A: 0.867 (0.818–0.916) B: 0.880 (0.799–0.961)	Calibration plot	Nomogram	—	DCA
Zhu Bifeng, 2024 (18)	Model development and validation	LR	Complete-case analysis	Categorical variables	Forward stepwise selection	Random split	Large arteries, TIA, blood glucose, Neu/Lym ratio, important perforator, ASPECTS	A: 0.809 (0.7429–0.8751) B: 0.816 (0.6783–0.9527)	Calibration plot	Nomogram	—	DCA
Qiu Kai, 2024 (19)	Model development and validation	LR	Complete-case analysis	Continuous variable	Univariate *p*-value	Random split	Age, symptom fluctuation characteristics, presence of core infarct, occlusion site	A: 0.930 (0.884–0.976) B: 0.889 (0.808–0.970)	H-L and calibration plot	Nomogram	—	DCA
Tian Tian, 2023 (23)	Model development and validation	LR	Complete-case analysis	Continuous variable	Backward stepwise selection	Cross validation and external validation	AF, BMI, NLR, MPV	A: 0.981 (0.961–1.000) B: 0.9815(0.9809–0.9821) C: 0.926 (0.868–0.985)	H-L and Brier score and calibration plot	Nomogram	—	DCA
Jin Huijuan, 2023 (24)	Model development and validation	LR	Complete-case analysis	Categorical variables	Stepwise selection	External validation	CK-MB, LDH, ALT, D-dimer, neutrophil ratio, NIHSS score, SBP	A: C-index 0.862 (0.796–0.928) C: C-index 0.900 (0.849–0.950)	H-L	Rating scale	—	—
Wu Kongyuan, 2023 (25)	Model development and validation	LR	Complete-case analysis	Categorical variables	Univariate *p*-value	Random split	Age, admission systolic blood pressure, initial NIHSS scores, history of hyperlipemia, location of occlusion	A: C-index 0.678 B: C-index 0.650	Calibration plot	Nomogram	—	DCA
Yang Huan, 2023 (26)	Model development and validation	LR	Complete-case analysis	Continuous variable	LASSO regression	Random split and 5-fold cross validation	Model 1: NIHSS score at admission, SBP, LYM% Model 2: NIHSS, SBP, LYM%, NLR, PNR, PLR	Model 1: A: 0.721 (0.651–0.792) B: 0.635 (0.518–0.752) Model 2: A: 0.862 (0.806–0.917) B: 0.816 (0.717–0.915)	H-L and calibration plot	Nomogram	—	—
Jin Mengzhi, 2023 (27)	Model development and validation	LR	Imputation	Continuous variable	Univariate *p*-value	Bootstrapping method	Endh: Stroke history, AF history, baseline NIHSS, ALT Endn: SBP, NIHSS, LAO	Endh: 0.934 (0.876–0.992) Endn: 0.811 (0.710–0.912)	Calibration plot	Nomogram	—	—
Yang Tongtong, 2023 (28)	Model development and validation	XGBoost, RF, LR, SVM	Complete-case analysis	Continuous variable	LASSO regression	Random split and 10-fold cross validation and external validation	Blood glucose, NIHSS at baseline, interval from groin puncture to recanalization, serum creatinine, interval from onset to treatment, systolic blood pressure, diastolic blood pressure, platelets, uric acid	A: XGBoost: 0.885 SVM: 0.642 RF: 0.856 LR: 0.655 B: XGBoost 0.826 (0.781–0.871) SVM: 0.643 (0.584–0.702) RF: 0.819 LR: 0.644 C: XGBoost: 0.846	Brier score	—	XGBoost	—
Wang Mei, 2023 (29)	Model development and validation	LR	—	Continuous variable	LASSO regression	Bootstrapping method	Post-thrombolysis NIHSS, pre-thrombolysis SBP, complication with atrial fibrillation, blood albumin	A: 0.796 (0.738–0.853) B: 0.785 (0.727–0.845)	Calibration plot	Nomogram	—	DCA
Wang Jia, 2022 (30)	Model development and validation	LR	—	Continuous variable	LASSO regression	Bootstrapping method	CRP, monocytes, NIHSS, SIRI	A: C-index 0.757 (0.702–0.805) B: C-index 0.747	Calibration plot	Nomogram	—	DCA
Xie Xiaohua, 2021 (31)	Model development	LR	Complete-case analysis	Continuous variable	Univariate *p*-value	—	Initial NIHSS score, MCA stenosis, carotid stenosis of ≥50%	0.870 (0.813–0.911)	Calibration plot	Nomogram	—	—
Seners Pierre, 2021 (32)	Model development and validation	LR	—	Categorical variables	Stepwise regression	Bootstrapping method and External validation	Occlusion site, thrombus length	A: C-index:0.76 (0.70–0.82) B: C-index: 0.75 (0.69–0.82) C: C-index: 0.78 (0.70–0.86)	H-L	Risk prediction score	—	—
Sung Sang Min, 2020 (33)	Model development	Boosted trees, Bootstrap decision forest, DNN, LR	—	Continuous variable	—	—	Hemorrhagic transformation, initial NIHSS score, stenosis of relevant artery, occlusion of relevant artery	Boosted trees: 0.934 Bootstrap decision forest: 0.932 DNN: 0.904 DLR: 0.885	—	—	Boosted trees	—
Gong Pengyu, 2020 (34)	Model development and validation	LR	Complete-case analysis	Continuous variable	Univariate *p*-value	External validation	AF, DM, Hs-CRP, NIHSS, age, previous antiplatelet medication	A: C-index 0.826 (0.785–0.885) C: C-index 0.798 (0.749–0.847)	Calibration plot	Nomogram	—	DCA
Xu Yicheng, 2020 (35)	Model validation	LR	Complete-case analysis	Continuous variable	Univariate *p*-value	External validation	An initial NIHSS score ≥8, diameter of infarction, striatocapsular infarction, TOAST type of large arterial atherosclerosis	0.80 (0.75–0.84)	—	—	—	—
Girot Jean-Baptiste, 2020 (36)	Model development	LR	Complete-case analysis	Continuous variable	Backward stepwise selection	—	Diabetes mellitus, prestroke modified Rankin Scale score ≥2, general anesthesia, admission SBP, age, number of passes, absence of direct patient admission to an EVT-capable center, lower pretreatment NIHSS score	C-index: 0.724	H-L	—	—	—
Miyamoto Nobukazu, 2017 (37)	Model development and validation	LR	Complete-case analysis	Categorical variables	—	External validation	Wrong (poor) blood sugar control [an HbA1c (NGSP) level of >7.4%]; old myocardial infarctions, ICA occlusion, MCA M1 occlusion, striatocapsular infraction, pontine infarction, the size of the infarct (15–30 mm), an elevated LDL cholesterol level (>140 mg/dL), neurological findings (an initial NIHSS score of > 8)	group 1: 0.789 group 2: 0.834	—	Risk prediction score	—	—

“—”, not reported; A, the training cohort; AF, atrial fibrillation; ALT, alanine transaminase; ANN, artificial neural network; ASPECTS, Alberta Stroke Program Early CT Score; AUC, area under the curve; B, the internal validation cohort; BMI, body mass index; C, the external validation cohort; CAD, coronary heart disease; C-index, concordance index; CK-MB, creatine kinase myocardial band; CRP, C-reactive protein; DBP, diastolic blood pressure; DCA, decision curve analysis; DM, diabetes mellitus; DNN, deep neural network; EVT, endovascular treatment; GBDT, Gradient Boosting Decision Tree; GBM, Gradient Boosting Machine; HbA1c, glycated hemoglobin; HCY, homocysteine; H-L, Hosmer-Lemeshow; Hs-CRP, hyper-sensitive C-reactive protein; ICA, internal carotid artery; LAO, large artery occlusion; LASSO, Least Absolute Shrinkage and Selection Operator; LDH, lactate dehydrogenase; LDL, low-density lipoprotein cholesterol; LR, logistic regression; LYM, lymphocyte; MCA, middle cerebral artery; MLP, multilayer perceptron; MPV, mean platelet volume; mRS, modified Rankin Scale; NGSP, National Glycohemoglobin Standardization Program; NIHSS, National Institutes of Health Stroke Scale; NLR, neutrophil-to-lymphocyte ratio; NNET, neural network; ONT, onset-to-needle time; OTT, onset-to-treatment time; PLR, platelet-to-lymphocyte ratio; PLS, partial least squares; PNR, platelet-to-neutrophil ratio; RF, Random Forest; SBP, systolic blood pressure; SII, Systemic immune-inflammation index; SIRI, systemic inflammatory response index; SVM, support vector machine; TBil, total bilirubin; TG, tree bag; TG, triglyceride; TIA, transient ischemic attack; TOAST, Trial of ORG 10172 in Acute Stroke Treatment; UA, uric acid; XGBoost, eXtreme Gradient Boosting.

### 3.4 Risk of bias and applicability assessment

**Figure 3:**
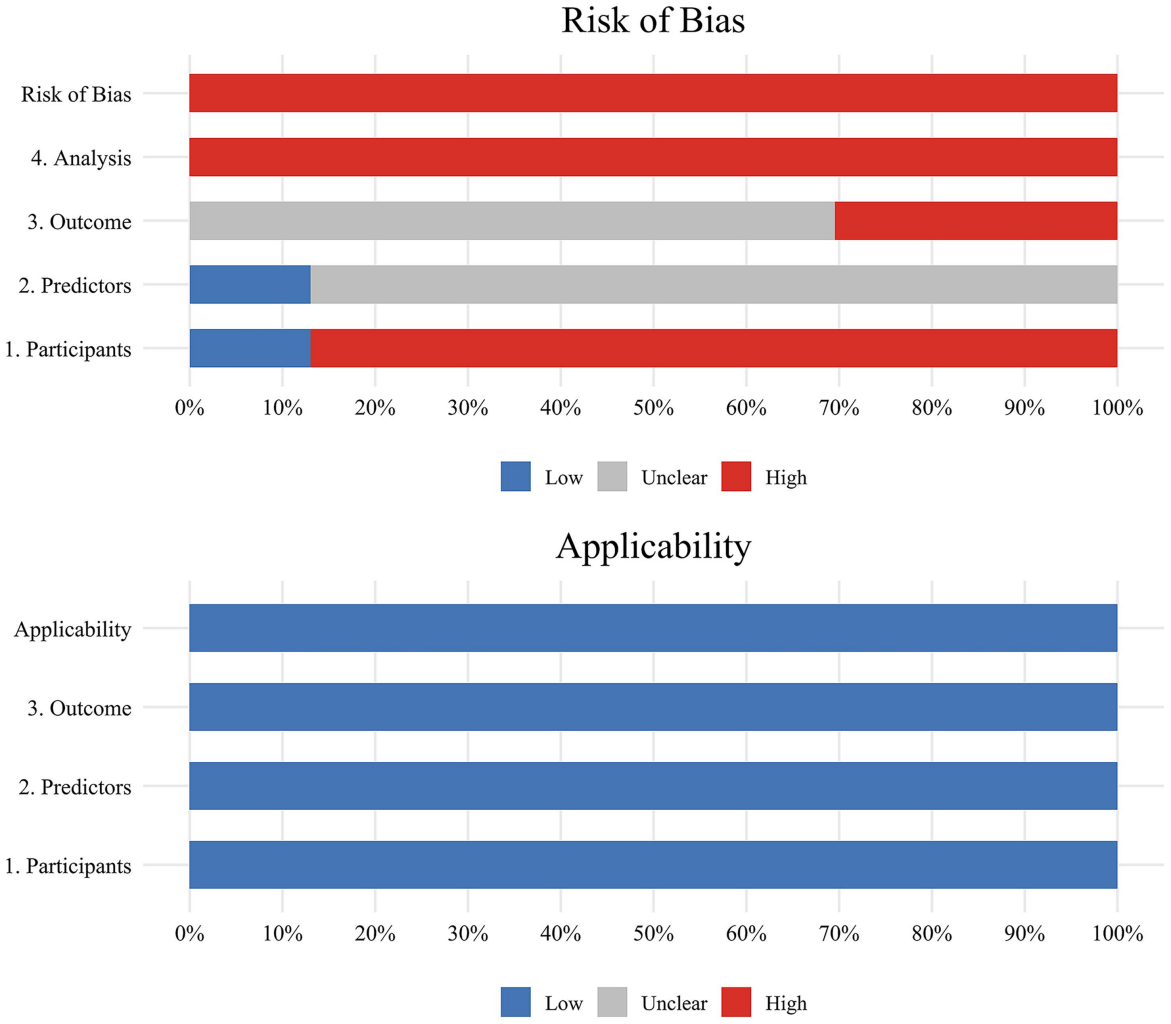
Graphical presentation of the ROB and applicability assessment based on PROBAST.

The overall and domain-specific ratings for the ROB and applicability of the 23 included studies are reported in Table 4 and Figure 3. All studies were found to have a high ROB, indicating several methodological problems in the model development or validation process.

**Table 4:** PROBAST results of the included studies.

First author, publication year	Study type	ROB				Applicability			Overall	
		Participants	Predictors	Outcome	Analysis	Participants	Predictors	Outcome	ROB	Applicability
Jiang Zhuangzhuang, 2024 (21)	B	−	?	?	−	+	+	+	−	+
Wang Jia, 2024 (22)	B	−	?	−	−	+	+	+	−	+
Luo Bang, 2024 (15)	B	−	?	−	−	+	+	+	−	+
Zhou Yang, 2024 (16)	B	−	?	?	−	+	+	+	−	+
Wen Rui, 2024 (17)	B	−	?	−	−	+	+	+	−	+
Li Ning, 2024 (20)	B	−	?	?	−	+	+	+	−	+
Zhu Bifeng, 2024 (18)	B	+	+	?	−	+	+	+	−	+
Qiu Kai, 2024 (19)	B	−	?	?	−	+	+	+	−	+
Tian Tian, 2023 (23)	B	−	?	?	−	+	+	+	−	+
Jin Huijuan, 2023 (24)	B	−	?	−	−	+	+	+	−	+
Wu Kongyuan, 2023 (25)	B	−	?	−	−	+	+	+	−	+
Yang Huan, 2023 (26)	B	−	?	−	−	+	+	+	−	+
Jin Mengzhi, 2023 (27)	B	−	?	−	−	+	+	+	−	+
Yang Tongtong, 2023 (28)	B	−	?	−	−	+	+	+	−	+
Wang Mei, 2023 (29)	B	−	?	−	−	+	+	+	−	+
Wang Jia, 2022 (30)	B	+	+	−	−	+	+	+	−	+
Xie Xiaohua, 2021 (31)	A	−	?	−	−	+	+	+	−	+
Seners Pierre, 2021 (32)	B	−	?	?	−	+	+	+	−	+
Sung Sang Min, 2020 (33)	A	−	?	−	−	+	+	+	−	+
Gong Pengyu, 2020 (34)	B	−	?	−	−	+	+	+	−	+
Xu Yicheng, 2020 (35)	C	−	?	−	−	+	+	+	−	+
Girot Jean-Baptiste, 2020 (36)	A	+	+	−	−	+	+	+	−	+
Miyamoto Nobukazu, 2017 (37)	B	−	?	−	−	+	+	+	−	+

PROBAST, Prediction model Risk Of Bias Assessment Tool; ROB, risk of bias. A indicates “development only”; B indicates “development and validation in the same publication”; C indicates “validation only.” + indicates low ROB/low concern regarding applicability; − indicates high ROB/high concern regarding application; and? indicates unclear ROB/unclear concern regarding applicability.

In the participant domain, 20 studies had a high ROB mainly owing to inappropriate data sources. In the predictor domain, 20 studies had an unclear ROB since they did not report information on blinding of predictor assessment to outcome data.

In the outcome domain, 16 studies were determined to have a high ROB, and seven studies were unclear (16, 18–21, 23, 32). Of these, 16 studies failed to exclude predictors from the outcome definition, all studies lacked both the indication of blind assessment and the mention of an appropriate time interval between predictor assessment and outcome determination.

In the analysis domain, all studies were found to have a high ROB. Ten studies demonstrated statistically inadequate sample size (15, 16, 18, 19, 21–24, 27, 28), failing to achieve the minimum requirement of 20 events per variable (EPV) as established in methodological guidelines. Six studies converted continuous variables into categorical variables without an appropriate explanation (18, 20, 24, 25, 32, 37). Only two studies included enrolled participants or used imputation of missing values (17, 27). Four studies did not specify the handling of missing data (29, 30, 32, 33). Fifteen studies did not avoid selecting variables based on univariate analysis. None of the studies provided information regarding complexities in the data. Four studies partially evaluated the predictive performance of their models (17, 33, 35, 37). Eleven studies did not fully account for model overfitting and performance optimism (15–22, 25, 34, 37). All studies fell short of providing sufficient details about the parameters of their predictive model, especially the predictors and regression coefficients of both the presented final model and the final multivariable analysis.

Despite the absence of studies with low ROB, applicability concerns were mitigated, as the target populations, predictors, and outcomes in the primary studies aligned with those specified in the review question.

## 4 Discussion

### 4.1 Main findings and interpretation

In this systematic review of prediction models for END in AIS, we identified 23 studies describing 45 models. We aimed to synthesize the published evidence on their predictive performance, methodological quality, and clinical applicability. The reported AUCs or C-statistics revealed remarkable heterogeneity in predictive accuracy across studies. All studies were appraised to have a high ROB owing to inappropriate data sources and poor reporting of the analysis domain. Concerns regarding applicability were generally low across studies, since the target setting and study population were described in detail.

The high ROB, inadequate model evaluation methods, heterogeneous predictive performance, insufficient diversity of patient populations, and unresolved potential for confounding variables in existing END prediction models are not isolated phenomena but interrelated elements forming a progressive causal relationship. Insufficient diversity of patient populations and inadequate consideration of confounding variables are fundamental root causes. They directly induce high ROB and inadequate model evaluation methods through data quality flaws and methodological loopholes, ultimately resulting in significant heterogeneity in model performance across different studies.

Insufficient diversity of patient populations is prominently reflected in the included studies. Geographically, 19 out of 23 studies were conducted in China, with only one each from Japan (37), Korea (33), and France (36), and one joint study from France and Switzerland (32), showing a high degree of regional concentration. This imbalance leads to model development based solely on the genetic background, healthcare system characteristics, and clinical practice patterns of specific ethnic groups. However, significant differences exist in the prevalence of risk factors such as atrial fibrillation and hypertension, as well as the accessibility and timing of reperfusion therapy, across different countries and ethnicities, directly affecting the adaptability of models in diverse populations. In terms of study population scope, nine studies focused exclusively on AIS patients receiving IVT (15, 17, 18, 20, 23, 24, 26, 27, 29), two on those undergoing MT (25, 28), and four on specific AIS subtypes (21, 22, 32, 33). This narrow focus limits models to specific subgroups, failing to cover patients ineligible for reperfusion therapy due to contraindications or other etiological types, which further restricts the generalizability of the models.

The inadequate consideration of potential confounding variables in model predictions also lays the groundwork for model flaws, with causes closely related to outcome definition, understanding of pathogenic mechanisms, and methodological processing. Regarding treatment-related confounding factors, among studies focusing on IVT patients, only a few included variables directly influencing END risk, such as onset-to-needle time (17, 18, 20, 24, 26, 27, 29), door-to-needle time (20, 26, 29), thrombolytic drug dosage, and post-thrombolysis antiplatelet/anticoagulant therapy (17). In MT-related studies, key treatment variables like puncture-to-recanalization time (28) and the number of recanalization attempts (25) were not consistently included as predictors. This omission stems from incomplete definition of END in some studies, which failed to exclude the overlap between predictors and outcome indicators, and excessive focus on baseline indicators in research design, neglecting the impact of treatment process variables, directly distorting the true association between other predictors and END outcomes. For physiological, laboratory, and temporal confounding factors, while common indicators such as baseline NIHSS score and SBP were widely incorporated, important confounders including renal function, electrolyte imbalances, and inflammatory markers beyond NLR were rarely considered. This is related to the current limited understanding of the pathogenic mechanisms of END, and the collection of some indicators requires additional laboratory tests, with insufficient data integrity in retrospective studies leading to their exclusion. Additionally, predictor assessment was mostly performed at admission, while outcome determination was concentrated 24 h to 7 days post-admission or treatment. Changes in patients’ clinical status, implementation of additional interventions, or occurrence of new complications during this period could not be captured by baseline data, further introducing confounding bias. Methodologically, fifteen studies selected variables based on univariate analysis, which may miss confounders with weak individual associations but significant interactive effects; six studies converted continuous variables to categorical variables without reasonable explanation (18, 20, 24, 25, 32, 37), obscuring potential linear or non-linear relationships between confounders and outcomes. These defects are partly due to the failure of some studies to strictly follow methodological guidelines such as TRIPOD, especially in early studies.

These two fundamental root causes directly induce high ROB through data quality flaws and methodological loopholes. Due to insufficient patient population diversity, single-center and homogeneous study populations lead to limited sample representativeness and inherent selection bias, which is the main reason why 20 studies were assessed as having a high ROB in the participant domain. The inadequate consideration of confounding variables results in information bias in variable selection and processing. Combined with methodological flaws such as 10 studies failing to meet the minimum requirement of 20 EPVs and most studies using complete-case analysis to handle missing data, all included studies were ultimately evaluated as having a high ROB. Meanwhile, these two root causes also directly lead to inadequate model evaluation methods: the lack of population diversity makes external validation across populations and centers unfeasible, resulting in 20 models undergoing only internal validation (15, 18–21, 25–27, 29, 30) and 6 models not being validated at all (31, 33, 36); the omission and improper handling of confounding variables mean most models can only focus on discrimination metrics such as AUCs and C-statistics, with only 13 studies assessing clinical utility through DCA, failing to fully reflect the models’ application value in real clinical settings.

The combined effect of high ROB and inadequate model evaluation methods ultimately leads to significant heterogeneity in model performance across different studies. From the perspective of model performance data, the AUC values of models in the derivation set ranged from 0.487 to 0.998, and from 0.493 to 0.982 in the validation set, showing substantial differences. We further stratified the comparisons by modeling approaches. Logistic regression-based models generally demonstrated stable performance across studies, while certain machine learning (ML) models (e.g., gradient boosting machines, random forests) achieved higher discriminative ability in specific datasets (AUC up to 0.88–0.93). However, some ML models performed poorly (AUC < 0.60), indicating that their performance heavily depends on data quality, feature engineering, and hyperparameter tuning. Notably, in external validation, traditional logistic regression models often showed better generalizability, whereas complex ML models exhibited greater performance fluctuation in unseen data.

The above analysis clearly reveals that the methodological limitations of existing END prediction models mainly focus on three core dimensions: patient population diversity, confounding variable control, and model evaluation system. Among these, insufficient patient population diversity and inadequate addressing of confounding variables are fundamental flaws that run through the entire process. Through data quality defects and methodological loopholes, they directly induce high risk of bias and an imperfect model evaluation system, ultimately leading to significant heterogeneity in predictive performance. Essentially, this reflects a mismatch between model development and clinical practical needs, which urgently requires breakthroughs through systematic methodological optimization.

Based on these identified limitations and core pain points, the following section will combine specific challenges faced by current research (such as the single method for sample size estimation and improper handling of missing data) with the latest methodological advancements and technical trends. It will propose targeted optimization pathways from dimensions including sample construction, variable processing, validation and updating, and technical application, providing practical guidance for the standardized development of high-quality END prediction models in the future and laying the foundation for unlocking the application potential of models in risk stratification and clinical decision-making for AIS patients.

### 4.2 Challenges and opportunities

Sample size estimation constitutes a pivotal methodological aspect in prediction model development and validation. Researchers have primarily relied on the EPV metric to determine sample size requirements. An adequate EPV can thus prevent overestimation of the model’s predictive performance (11). To mitigate overfitting risks, a minimum EPV threshold of 10 has been empirically established and widely endorsed in statistical practice (38, 39). Furthermore, various authors have recommended that higher EPVs of at least 20 may be warranted to ensure robust model development (40). However, the EPV treats each predictor as a single unit regardless of its type and often ignores the complexity of predictors, such as continuous variables modeled nonlinearly or categorical variables with three or more categories, which require numerous parameters. By concentrating on the number of candidate predictor parameters (i.e., *β* terms) rather than just the number of predictors, the events per predictor parameter (EPP) offers a more sophisticated and accurate method for developing multivariable prediction models. It takes predictor complexity into account to prevent sample size underestimation and guarantee model stability and reliability (41). A key concern is that blanket rules of thumb for sample size determination are known to be overly simplistic. To address this, a range of mathematically rigorous sample size calculation methods have been developed, alongside dedicated software tools to facilitate their implementation (41–44). When the available dataset’s sample size fails to meet the minimum requirements for model development or external validation, the recommended approach is to reduce the number of candidate predictor parameters rather than compromising on model quality (45).

Missing data are often inadequately handled and reported in clinical prediction model research (46). Information on missing data should be reported as part of the results of the studies. Complete-case analysis, which is the most common approach to handling missing data, deletes individuals with missing data on any predictor or outcome variables. This method is likely to cause bias and reduce analytical power in both prediction model development and predictive accuracy estimates (47). Therefore, multiple imputation strategies are recommended to handle missing data (47, 48).

In recent years, prediction models for END in AIS have proliferated, yet a striking imbalance persists: innovative model development is fervently pursued, while systematic external validation and dynamic updating remain scarce. In our study, 20 models performed internal validation only, and six models were not validated after development (31, 33, 36). This not only compromises the portability and generalizability of the models (49, 50), but also results in duplicate research efforts and resource wastage. To break this cycle, external validation and continuous updates must be given the same weight as model development itself. The model must first undergo external validation in independent, distinct populations; otherwise, its application in a new healthcare setting or country will likely result in miscalibrated predictions (51). Adopting this discipline will substantially enhance the applicability and robustness of prediction models in routine clinical settings.

Machine learning (ML) is a field of study in which computers are enriched with the capability of acting without being explicitly programmed (52). During the past decade, advances in artificial intelligence (AI), especially in ML, have reshaped clinical decision-making (53, 54). Numerous studies have revealed that ML models are crucial for predicting the prognosis and treatment effectiveness of neurological disorders (55, 56). However, ML models in our study did not outperform traditional methods because some algorithms yielded low AUCs in both the training and the validation sets (21). When choosing the model type, one should base it on clinical goals, data quality and interpretability requirements. A trade-off should be made between simple and robust logistic regression and high-performance but strictly validated machine learning models. Ultimately, clinical applicability should be the core decision criterion. Notably, the PROBAST assessment framework may not be fully applicable to the evaluation of ML models because its design logic and evaluation dimensions are structurally at odds with the ML modeling process. Therefore, it is recommended to adopt the emerging frameworks PROBAST-AI (57) and TRIPOD-AI (58) as the primary guiding tools for reporting and evaluating AI/ML-based predictive models in the future.

In clinical practice, the clinical utility of predictive models hinges on three core pillars: user-friendly presentation formats (59), interpretable decision support, and reasonable cost-effectiveness. Comprehensive reporting of model parameters (e.g., regression coefficients, cut-off values) lays the foundation for external validation or model updates by other researchers, while more accessible formats such as websites and applications can significantly enhance the model’s applicability in routine clinical settings. Of particular importance is that model interpretability serves as a key prerequisite for improving clinical trust. When predicting the recurrence of spontaneous intracerebral hemorrhage using machine learning models, Cui et al. achieved feature importance visualization and individual prediction process explanation through tools like SHAP (SHapley Additive exPlanations) and LIME (Local Interpretable Model-agnostic Explanations) (60). Meanwhile, cost-effectiveness is a critical consideration for the widespread application of models. Our study involves a large number of predictive factors, some of which are difficult to promote due to complex technologies or high costs. However, for routinely collected clinical indicators, data-driven methods (e.g., unsupervised clustering) may be valuable in exploring new associations between these indicators and outcomes (61), but this is not applicable to factors that are inherently difficult or expensive to obtain. Therefore, the final selection of predictive factors should be based on robust clinical evidence. In studies with limited samples, while data-driven methods can be used for initial exploration, their findings must not be directly adopted as screening criteria; instead, they must be rigorously confirmed through independent prospective cohort validation and multivariate analysis. Expert consensus and existing literature play a crucial guiding and evaluating role in this validation process (62). As routine imaging modalities for AIS, CT, and MRI contain abundant imaging features that should be thoroughly analyzed and converted into effective predictive indicators. Additionally, easily accessible biomarkers such as tongue images and facial features are being integrated into disease screening systems through emerging technologies (63, 64). Such indicators can improve model utility without increasing clinical burden, making them particularly suitable for resource-limited settings. Looking forward, models should evolve from risk warning to intervention guidance. By integrating multimodal data to predict patients’ benefits from targeted interventions, while actively addressing implementation challenges such as data standardization and cross-institutional validation, the clinical application value of models can be further enhanced.

### 4.3 Study limitations

The limitations of this analysis need to be acknowledged. First, due to the high heterogeneity observed in the selected population and the model characteristics, it is inappropriate to carry out the meta-analysis, and only a narrative synthesis was conducted. Second, we included studies only published in English, so relevant studies in other languages may have been omitted.

## 5 Conclusion

This systematic review provides a comprehensive mapping and critical assessment of existing END prediction models in AIS. The findings reveal a critical gap that current models exhibit high ROB, limiting their reliability for clinical adoption. Future research should prioritize prospective model development and validation with pre-specified protocols, rigorous adherence to methodological standards such as the TRIPOD guidelines, adequate sample size estimations, robust external validation, as well as the update and clinical utility of existing predictive models.

## Data Availability

The original contributions presented in the study are included in the article/Supplementary material, further inquiries can be directed to the corresponding authors.
