# Supplementary material for: Prediction models for early neurological deterioration in patients with acute ischemic stroke: a systematic review and critical appraisal
Source: Front Neurol. 2026 Feb 24;17:1737871. doi: 10.3389/fneur.2026.1737871 (PMC12971439; doi:10.3389/fneur.2026.1737871)
Supplement: Supplementary file 1 [file Table_1.docx]

Supplementary Material

**1 Supplementary data**

For retrieval of databases, the keywords of search are the same, with taking PubMed as an example:

**((((((((((((((Ischemic Stroke[Title/Abstract]) OR (Stroke, Ischemic[Title/Abstract])) OR (Ischaemic Stroke[Title/Abstract])) OR (Acute Ischemic Stroke[Title/Abstract])) OR (Ischemic Stroke, Acute[Title/Abstract])) OR (Stroke, Acute Ischemic[Title/Abstract])) OR (Ischemic cerebrovascular disease[Title/Abstract])) OR (Brain Ischemia[Title/Abstract])) OR (Ischemic Encephalopathy[Title/Abstract])) OR (Cerebral Ischemia[Title/Abstract])) OR (Cerebral Infarction[Title/Abstract])) OR (Brain Infarction[Title/Abstract])) OR (cerebral embolism[Title/Abstract])) AND ((((((((((Nomogram[Title/Abstract]) OR (development[Title/Abstract])) OR (validation[Title/Abstract])) OR (prediction[Title/Abstract])) OR (prediction model[Title/Abstract])) OR (predictive model[Title/Abstract])) OR (risk score[Title/Abstract])) OR (risk scale[Title/Abstract])) OR (risk prediction[Title/Abstract])) OR (risk assessment[Title/Abstract]))) AND ((((((Neurological deterioration[Title/Abstract]) OR (neurologic deterioration[Title/Abstract])) OR (neurological worsening[Title/Abstract])) OR (neurologic worsening[Title/Abstract])) OR (neurological decline[Title/Abstract])) OR (neurological change[Title/Abstract]))**
